# Effects of Three-Month Feeding High Fat Diets with Different Fatty Acid Composition on Myocardial Proteome in Mice

**DOI:** 10.3390/nu13020330

**Published:** 2021-01-23

**Authors:** Adam Lepczyński, Małgorzata Ożgo, Katarzyna Michałek, Alicja Dratwa-Chałupnik, Marta Grabowska, Agnieszka Herosimczyk, Kamila P. Liput, Ewa Poławska, Andrzej Kram, Mariusz Pierzchała

**Affiliations:** 1 Department of Physiology, Cytobiology and Proteomics, West Pomeranian University of Technology, K. Janickiego 32 Str., 71-270 Szczecin, Poland; malgorzata.ozgo@zut.edu.pl (M.O.); katarzyna.michalek@zut.edu.pl (K.M.); alicja.dratwa-chalupnik@zut.edu.pl (A.D.-C.); agnieszka.herosimczyk@zut.edu.pl (A.H.); 2 Department of Histology and Developmental Biology, Pomeranian Medical University, Żołnierska 48, 71-210 Szczecin, Poland; martag@pum.edu.pl; 3 Department of Molecular Biology, Institute of Genetics and Animal Biotechnology of the Polish Academy of Sciences, Postepu 36A Str., Jastrzebiec, 05-552 Magdalenka, Poland; k.stepanow@igbzpan.pl; 4 Department of Genomics and Biodiversity, Institute of Genetics and Animal Biotechnology of the Polish Academy of Sciences, Postepu 36A Str., Jastrzebiec, 05-552 Magdalenka, Poland; e.polawska@igbzpan.pl (E.P.); m.pierzchala@igbzpan.pl (M.P.); 5 Department of Pathology, West Pomeranian Oncology Center, Strzałowska 22 Str., 71-730 Szczecin, Poland; akram@onkologia.szczecin.pl

**Keywords:** proteomics, high fat diet, saturated fatty acids, polyunsaturated fatty acids, cardiac muscle, omega-6/omega-3 ratio

## Abstract

Westernized diet is characterized by a high content of saturated fatty acids (SFA) and a low level of omega-3 polyunsaturated fatty acids (PUFA), often accompanied by an imbalance in the omega-6/omega-3 PUFA ratio. Since increased intake of SFA and n-6 PUFA is considered as a cardiovascular disease risk factor, this study was conducted to determine whether a three-month dietary supplementation of high-fat diets (HFDs) with saturated fatty acids and a significant proportion of various n-6 and n-3 PUFA ratios would affect the architecture and protein expression patterns of the murine heart. Therefore, three HFD (*n* = 6) feeding groups: rich in SFA, dominated by PUFA with the n-6/n-3–14:1, and n-6/n-3–5:1, ratios were compared to animals fed standard mouse chow. For this purpose, we performed two-dimensional electrophoresis with MALDI-ToF mass spectrometry-based identification of differentially expressed cardiac proteins, and a histological examination of cardiac morphology. The results indicated that mice fed with all HFDs developed signs of hypertrophy and cardiac fibrosis. Animals fed SFA-rich HFD manifested the most severe cardiac hypertrophy and fibrosis lesions, whereas less pronounced changes were observed in the group of animals that ingested the highest amount of omega-3 FA. In general, all HFDs, regardless of FA composition, evoked a comparable pattern of cardiac protein changes and affected the following biological processes: lipid metabolism and FA β-oxidation, glycolysis, TCA cycle, respiratory chain, myocardium contractility, oxidative stress and PUFA eicosanoid metabolism. However, it should be noted that three proteins, namely IDH3A, LDHB, and AK1, were affected differently by various FA contents. High expression of these myocardial proteins found in the group of animals fed a HFD with the highest n-3 PUFA content could be closely related to the observed development of hypertrophy.

## 1. Introduction

Obesity has become a pandemic of the 21st century and is undoubtedly one of the greatest public health challenges for both humans and companion animals. It has been estimated that if the current trend continues, presumably up to 36.6% of men and 32.0% of women in Europe will be either overweight or obese by 2030 [1]. High prevalence of obesity is one of the leading causes of elevated cardiovascular diseases (CVD), especially in Western societies [2,3]. It should be highlighted that CVD mortality is closely and directly related to the consumption of a nutrient-poor Western diet [4]. Westernized diet is generally defined as a high dietary intake of sugar, saturated fatty acids (SFA), and n-6 polyunsaturated fatty acids (PUFA), with associated reduced consumption of n-3 PUFA and fibre [5,6]. Polyunsaturated fatty acids (PUFA) belong to the group of the so-called essential fatty acids as mammals cannot synthesize n-3 and n-6, and thus they must be supplied with food [7]. N-6 and n-3 PUFA are bioactive compounds that exert a profound impact on various physiological processes [8]. Increased n-6 fatty acids intake, which is observed along with modified dietary patterns, induces changes in the n-6/n-3 ratio. Currently, this ratio is 15:1 in favor of n-6 acids, and it differs significantly from the diet of our ancestors, in which this ratio was close to one. Currently, the recommended dietary ratio of these fatty acids (FAs) is 2–5:1 [6,9]. This is based on the fact that there is a competition between fatty acids of n-6 and n-3 series as both of these FAs are metabolized by the same group of enzymes to their respective metabolites. Since n-6 acids are the most preferred substrates for those enzymes, their higher dietary content dramatically reduces the utilisation of n-3 acids. In addition, there is evidence suggesting that many gene-defined enzymes and receptors favor n-6 mediators and cause physiological and pathophysiological effects [10]. As a result of n-6 fatty acid intake, significant quantities of arachidonic acid (AA) and its active metabolites (PGE2 and PGI2 prostaglandins, TXA2 thromboxane, A4, B4, C4, D4, and E4 leukotrienes) are generated, which, unlike n-3 acid metabolites [11], induce inflammatory processes, show a thrombotic effect as well as enhance the synthesis of free radicals. The results of numerous in vivo and in vitro studies have clearly indicated that n-6 acids may also directly influence myocardial function. For example, AA-derived mediators have been shown to significantly affect cardiac arrhythmogenesis [12]. Furthermore, Fluri et al. (1990) and Schmilinsky-Fluri et al. (1997) found that AA had the ability to induce changes in the integrity of gap junctions between cardiac myocytes [13,14]. A higher ratio of dietary n-6/n-3 fatty acids (FAs) may also result in the activation of pro-inflammatory metabolic pathways in the myocardium, causing the generation of significant amounts of reactive nitrogen and oxygen species [15]. It has also been proven that dietary linoleic acid (LA) and its metabolites can induce collagen synthesis by cardiac fibroblasts, which can lead to fibrosis and increased stiffness of the left ventricle [16].

Considering the above, we hypothesized that feeding mice for three months high-fat diets with saturated fatty acids and a significant proportion of different ratios of unsaturated n-6 and n-3 fatty acids would affect the mouse heart architecture and the expression of proteins involved in cardiac muscle contraction, energetic metabolism, and stress response. 

Therefore, the main purpose of this study was to (1) analyse the cardiac muscle histology, and (2) to screen for differences in protein expression patterns in the hearts of mice fed three high-fat diets with different fatty acid compositions, including SFA and PUFA with two different n-6 to n-3 ratios (14:1 and 5:1, respectively).

## 2. Materials and Methods 

### 2.1. Animals, Diets, Housing Conditions, and Experiment Termination

The nutritional experiment was conducted in the vivarium of the Institute of Genetics and Animal Biotechnology of the Polish Academy of Sciences in Jastrzębiec. Experimental procedures were approved by the II Warsaw Local Ethics Committee for Animal Experimentation (WAW2_22/2016). Animals were maintained in standard cages under temperature- and humidity-controlled conditions with a 12-h light/dark cycle. Animals received water and food *ad libitum*. 

Male Swiss-Webster mice (*n* = 24) were fed standard growth diets for 8 weeks after weaning. Next animals were randomly selected into four dietary groups (*n* = 6) that were fed with appropriate diet. Animals of the control group (STD) were fed with standard chow for mice Labofeed H (Morawski, Żurawia, Poland). Experimental groups were fed high fat diets. Animals of first experimental group (SFA group) were fed a diet rich in saturated fatty acids (SFA group), which was composed with the addition of coconut virgin oil. Two other diets were dominated by the PUFA with different linoleic acid (LA) to α-linolenic acid (ALA) ratio. The diet with the high n-6 to n-3 FA ratio was prepared on the basis of the standard chow with addition of pumpkin seed oil. It allowed to obtain the diet with the n-6/n-3 ratio equal 13.76:1, which was used for feeding the 14:1 group. The last group of animals (5:1 group) were fed with a diet enriched with different vegetable oils that after the addition to the standard chow resulted in the n-6/n-3 ratio close to 5:1. Oil additives level in the experimental diets are given in Table 1. Confirmation of FA composition in used oils were estimated using a GC-7890 gas chromatograph (Agilent Technologies, Inc., Santa Clara, CA, USA) with a flame ionization detector (FID) and a 60 m capillary column, 0.25 mm internal diameter and 0.20 μm stationary layer thickness (Hewlett-Packard-88, Agilent J&W GC Columns, Santa Clara, CA, USA). Helium with a flow rate of 50 mL/min was used as a carrier gas. The dispenser and detector temperature was 260 °C. Temperature program: (1) from 140 °C to 190 °C (4 °C/min), (2) from 190 °C to 215 °C (0.8 °C/min). Supelco 37 Component FAME Mix, 47885-U (Sigma-Aldrich Co., St. Louis, MO, USA) standards were used for the FA determination. The total fat content in STD chow and SFA, 14:1 and 5:1 diets were about 2% and 22%, respectively. The diets were produced manually, divided in aliquots, vacuum packed, and stored in dark. Diets were given to the animals two times per day to avoid oxidation.

At the end of experiment after 12 h fasting period animals were euthanized in UNO Euthanasia Unit (Uno Roestvaststaal BV, Zevenaar, Netherlands) with 100% CO_2_ gas after the saturation of the blood with O_2_ by the exposition to carbogen. Immediately after the procedure the blood was collected via the heart puncture, and subsequently animal hearts were collected. 

### 2.2. Plasma Biochemistry

Blood was collected into the EDTA coated tubes by cardiac puncture and mixed with and centrifuged at 3000 rcf for 10 min at 4 °C to obtain blood plasma. Blood plasma was stored at −70 °C. Biochemical analyses were performed using COBAS INTEGRA^®^ 400 plus system (Roche Diagnostics Ltd., Rotkreuz, Switzerland) using ready prepared kits. 

### 2.3. Histological Analyses

Hearts obtained during the section were fixed in 4% buffered paraformaldehyde and were then embedded in paraffin blocks. Next, 3 µm sections were cut using a microtome and then were placed on the poly-lysine-coated slides. 

#### 2.3.1. Hematoxylin and Eosin Staining (H&E)

Hematoxylin and eosin staining (H&E) was performed according to a protocol described in detail by Gamble (2008) [17]. The heart tissue sections were deparaffinized and rehydrated. Next, the sections were first stained with Mayer’s hematoxylin for 5 min and washed in running water. Subsequently, the tissue sections were stained with eosin for 1 min and washed in distilled water. At the end, they were dehydrated and coverslipped. 

#### 2.3.2. Mallory Trichrome Staining

Hearts tissue sections after deparaffinization and rehydration were stained in 1% acid fuchsin solution (Sigma-Aldrich, St. Louis, MO, USA) in distilled water and next in 5% phosphotungstic acid solution for 20 min. At the next stage, the tissue sections were stained in a solution of 1% aniline blue (Sigma-Aldrich, St. Louis, MO, USA), 2% orange G (Sigma-Aldrich, St. Louis, MO, USA), and 2% oxalic acid (Sigma-Aldrich, St. Louis, MO, USA) in distilled water for 30 min. Afterwards, the slides were dehydrated and coverslipped.

#### 2.3.3. Quantitative Analysis of Mallory’s Trichrome Staining and Morphological Parameters

Using a ScanScope AT2 scanner (Leica Microsystems, Wetzlar, Germany) H&E-stained and Mallory’s trichrome-stained hearts tissue sections were subjected scanning procedure at magnification of 400× (resolution of 0.25 μm/pixel). Subsequently, the obtained digital images of the tissue sections were analyzed on the computer screen with the use an ImageScope viewer software (v. 11.2.0.780; Aperio Technologies, Inc., Vista, CA, USA).

The cardiomyocytes diameter (µm) were assessed using ruler tool on the H&E-stained heart tissue sections. In each group, one hundred sixty longitudinally sectioned cells in the nucleus region were analyzed (16 cells in each mouse). 

For the quantitative analysis of collagen on the Mallory’s trichrome-stained hearts tissue sections, a positive pixel count v9 algorithm (v. 9.1; Aperio Technologies, Inc., Vista, CA, USA) was used. Other parameters have been set to get compliance with the visual assessment of color intensity. The areas of the analysis were manually determined. The percent of collagen that was positive for Mallory’s trichrome staining were determined in 30 random fields for each group (5 in each mouse), with an average area of 1.25 mm^2^ (for STD group), 1.60 mm^2^ (for SFA group), 1.48 mm^2^ (for 14:1 group), and 1.56 mm^2^ (for 5:1 group). 

### 2.4. Two-Dimensional Electrophoresis

#### 2.4.1. Homogenization

After the collection hearts were washed in 0.9% NaCl solution and after that were weighted. Next, the hearts were frozen in the liquid nitrogen and stored in −80 °C until sample homogenisation. After defreezing in the presence of HEPES buffer, the hearts were opened using surgical blade and residual blood clots were washed out. Immediately after that whole hearts were pulverized using mortar in the presence of liquid nitrogen. Tissue powder was than homogenized using zircon beds (1.4 mm of diameter) using Tissue Lyser (Quiagene, Venlo, Netherlands) (20 min, 21 Hz) in lysis buffer containing 5 M urea, 2 M thiourea, 4% *w*/*v* CHAPS, and protease inhibitors (Protease Inhibitor Cocktail, Merc, St. Louis, MO, USA). Subsequently the homogenates were centrifuged at 20,800× *g* for 25 min at 4 °C. Harvested supernatants were stored at −80 °C.

#### 2.4.2. Isoelectrofocusing (IEF)

In heart protein samples total protein concentration was determined by a modified Bradford assay (Protein Assay Dye Reagent Concentrate; Bio-Rad, Bio-Rad, CA, USA). Protein samples containing 350 μg of total protein, in 250 μL of lysis buffer (5 M urea, 2 M thiourea, 4% CHAPS, 1% (*w*/*v*) dithiothreitol (DTT), 0.5% (*v*/*v*) carrier ampholytes). Each sample was than subjected for IEF. Prior IEF samples were loaded in 11 cm IPG strips with non-linear 3–10 pH gradient by in-gel strips via rehydratation process (passive—5 h, 0 V and active—12 h, 50 V). Subsequently isoelectric focusing was performed using Protean i12 IEF Cell (Bio-Rad, Hercules, CA, USA) in total of 37 kVh. Following IEF program was used: 250 V for 125 Vh, 500 V for 250 Vh, 1000 V for 500 Vh in rapid mode, linear voltage increase to 3500 V in 1:30 h, 3500 V for 35,000 Vh in rapid mode. 

#### 2.4.3. Second Dimension—SDS-PAGE 

Immediately after IEF the IPG strips were equilibrated for 15 min in basal buffer (6 M urea, 0.5 M Tris/HCl, pH 6.8, 2% *w*/*v* SDS, 30% *w*/*v* glycerol) with 1% DTT addition. Next, strips were washed for 20 min in the basal equilibration buffer with addition of 2.5% iodoacetamide. Second dimension of electrophoresis was run at 40 V for 2.5 h and subsequently at 100 V for 16 h (15 °C) in 12% polyacrylamide gels in Protean Plus™ Dodeca Cell™ electrophoretic chamber (Bio-Rad, Hercules, CA, USA). To allow protein molecular masses comparison the samples were co-run with Precision Plus Protein™ Kaleidoscope™ Standard for SDS-PAGE (Bio-Rad, Hercules, CA, USA) as a reference

#### 2.4.4. Image Staining and Analysis

After two dimensional electrophoresis (2-DE) separation, proteins in gels were detected with CBB G-250. The gels were placed in Dodeca™ Gel Stainer, large (Bio-Rad, Hercules, CA, USA) and washed with fixation buffer (50% ethanol, 5% phosphoric acid in ddH_2_O) for 3 h. Than the buffer was replaced with the ready stock Bradford solution (Bio-Rad Protein Assay, Bio-Rad, Hercules, CA, USA) diluted 20 times in ddH_2_O and gels were stain in that solution for 3 h. After staining, gels were washed in the ddH_2_O 3 times for 15 min. After staining, gels images were digitalized using a GS-800™ Calibrated Densitometer (Bio-Rad, Hercules, CA, USA).

Obtained gel images of cardiac proteome were analyzed using PDQuest Analysis software 8.0.1, Advanced (Bio-Rad, Hercules, CA, USA). To measure the variability within the group, the coefficient of variation (CV) was calculated for each experimental group. Qualitative and quantitative comparisons between the replicate groups were performed to highlight the significant differences in the protein expression pattern. Experiment normalization was performed using a local regression model (LOESS). The experimental isoelectric points (pI) and molecular weight (kDa) values were computed for each identified differentially expressed protein spot.

#### 2.4.5. Matrix-Assisted Laser Desorption Ionization—Time of Flight Mass Spectrometry (MALDI-ToF MS)

The protein spots that showed significantly differentiated expression were identified by peptide mass fingerprinting using MALDI-ToF mass spectrometer Microflex (Bruker, Brema, Germany). At least two biological replicates for each protein spots were manually excised from polyacrylamide gels. Then excised spots were destined in a buffer containing 25 mM NH4HCO3 in 5% *v*/*v* acetonitrile (ACN), followed by two washes with a solution of 25 mM NH4HCO3 in 50% *v*/*v* ACN. After each washing step protein spots were incubated at room temperature for 10 min in ultrasonic bath. Right after the decolorization, protein spots were dehydrated in 100% ACN for 20 min in ultrasonic bath and subsequently vacuum-dried for 15. The dry gel pieces were incubated with trypsin (20 µL/spot of 12.5 µg trypsin/mL in 25 mM NH4HCO3; Promega, Madison, WI, USA) at 37 °C as previously described by Ożgo et al. [18].

Resulting peptides were extracted with 100% CAN and combined using dry droplet method on the MALDI-MSP AnchorChip™ 600/96 plate (Bruker Daltonics, Brema, Germany) target with the matrix solution (5 mg/mL CHCA, 0.1% *v*/*v* TFA, 50% *v*/*v* ACN) in a final volume of 1 µL. The Microflex™ MALDI-TOF (matrix-assisted laser desorption/ionization time of flight) mass spectrometer (Bruker Daltonics, Brema, Germany) was operated in a positive ion reflector mode. External calibration was performed using Peptide Mass Standard II (Bruker Daltonics). The mass spectra were acquired with 150 shots of a nitrogen laser operating at 20 Hz and were internally calibrated using porcine tryptic autolytic products (842.51 and 2211.10 *m*/*z*). The mass spectra were acquired using the FlexControl 3.0 (Bruker, Brema, Germany) software and subsequently processed using the FlexAnalysis 3.0 (Bruker, Brema, Germany) software. Protein identification was performer using the Peptide Mass Fingerprinting (PMF) technique. Spectra were compared to mammalian SwissProt/NCBI databases using MASCOT search angine (http://www.matrixscience.com/). The following cryteria were used for database searches: (1) trypsin digestion with maximum one missed cleavage site; (2) cysteine carbamidomethylation as a fixed modification; (3) acetylation and methionine oxidation as variable modifications; (4) mass tolerance to 150 ppm. 

#### 2.4.6. Geneo Ontology Analyses

Cytoscape software presented biologic role specificity of most significantly differentially expressed gene products on the basis of their functional and pathway enrichment analyses including gene ontology databases: Genes in KEGG 08.05.2020-8024; Genes in WikiPathways 08.05.2020-293; Genes in INTERPRO_ProteinDomains 08.05.2020-12084; Genes in REACTOME_Reactions 08.05.2020-11188; Genes in GO_ImmuneSystemProcess-EBI-UniProt-GOA-ACAP-ARAP 08.05.2020_00h00-3426; Genes in REACTOME_Pathways 08.05.2020-10925; Genes in GO_MolecularFunction-EBI-UniProt-GOA-ACAP-ARAP 08.05.2020 00h00-17817; Genes in GO_CellularComponent-EBI-UniProt-GOA-ACAP-ARAP 08.05.2020 00h00-18937; Genes in GO_BiologicalProcess-EBI-UniProt-GOA-ACAP-ARAP 08.05.2020 00h00-17972. The *homo sapiens* was taken as an reference [19]. Differentially expressed gene products not involved in the CluGo analysis were categorized according to their biological functions and known pathways using STRING v. 11.0b [20]. Subcellular localisation of proteins were defined according to UniProtKB database (www.uniprot.org).

### 2.5. Western Blot

In the present study, the following primary antibodies were used to examine expression of selected proteins in the mouse heart: (1) mouse monoclonal anti- short-chain specific acyl-CoA dehydrogenase, ACADS (sc-365953, Santa Cruz Biotechnology, Santa Cruz, CA, USA); (2) mouse monoclonal anti- bifunctional epoxide hydrolase 2, sEH (sc-166961, Santa Cruz Biotechnology); (3) mouse monoclonal anti- superoxide dismutase 1, SOD1 (sc-101523, Santa Cruz Biotechnology, Santa Cruz, CA, USA); (4) mouse monoclonal anti-malate dehydrogenase, mitochondrial, MDH2 (sc-293474, Santa Cruz Biotechnology, Santa Cruz, CA, USA). Labeling of the antigen-antibody complexes were visualized with the use of secondary monoclonal goat anti-mouse (sc-516102, Santa Cruz Biotechnology, Santa Cruz, CA, USA) horseradish peroxidase-conjugated antibodies.

In the obtained supernatants the total protein was determined by the modified Bradford method (Protein Assay Dye Reagent Concentrate, Bio-Rad, Hercules, CA, USA). Subsequently, heart homogenates were mixed with the Leammli buffer in such proportions, so that after applying 10 μL of the sample to the wells, each of them contained 10 μg of total protein. The samples were warmed at 37 °C for 15 min and loaded on 12% polyacrylamide gels and run for 120 min at 100 V. Subsequently, the proteins were then electrotransfered (12 V, 14 min) from the gels to PVDF membranes. The membranes were blocked with 5% nonfat-milk in PBS-T (80 mM Na2HPO4, 20 mM NaH2PO4, 100 mM NaCl, and 0.1% Tween 20, pH 7.5) for 1 h and incubated overnight at 4 °C with the primary antibodies. In the current experiment, the following dilutions were used: anti-ACADS 1:500, anti-sHE 1:100, anti-SOD1 1:500, and anti-MDH2 1:100. The membranes were then incubated with a secondary anti-mouse horseradish peroxidase-conjugated antibody diluted 1:1000. The labeling was visualized by an enhanced chemiluminescence system (ECL Plus, Thermo Fisher Scientific, Waltham, MA, USA) and exposure to a CCD camera (Versadoc 4000MP, Bio-Rad, Hercules, CA, USA). The obtained images were recorded in a digital form and modified (auto-scale was used, speckles were removed and a representative band was cut out) using the Quantity One and PDQuest software (Bio-Rad, Hercules, CA, USA).

### 2.6. Statistic Analysis

The quantitative analysis of the differences in protein spot abundance, Student’s t-test was used as integrated in the PDQuest 8.0.1 software (Bio-Rad, Hercules, CA, USA). Significance of the differences was set at the level of *p* ≤ 0.05.

Biochemical data were analyzed by one-way analysis of variance. Differences between treatments were analysed by post hoc Tukey honestly significant. Differences at *p* < 0.05 were considered to be statistically significant.

The quantitative values for cardiomyocytes diameter and collagen were first analyzed for normality using the Shapiro–Wilk test. Because of the obtained values failed normal distribution assumption, the Kruskal–Wallis test with Dunn’s multiple comparison test for post hoc analysis was applied to compare the difference between the groups. Differences at *p* < 0.05 were considered to be statistically significant.

## 3. Results

### 3.1. Morphometric and Histological Heart Parameters and Blood Plasma Biochemistry 

**Figure 1:**
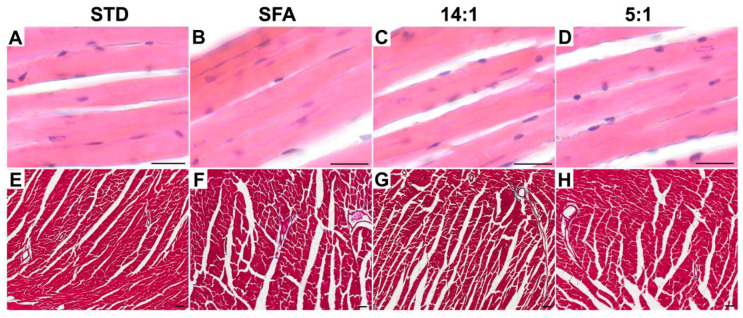
Representative light micrographs of H&E-stained (**A**–**D**) and Mallory’s trichrome-stained (**E**–**H**) ventricle mid-region heart cross sections after STD (**A**,**E**), SFA (**B**,**F**), 14:1 (**C**,**G**) and 5:1 (**D**,**H**) diets. Scale bar—50 µm. STD – standard diet; SFA – high fat diet rich in saturated fatty acids; 14:1 – high fat diet rich in polyunsaturated fatty acids (PUFA) with LA/ALA ratio ~14:1; 5:1 – high fat diet rich in (PUFA) with LA/ALA ratio 5:1.

None of HFDs affected murine heart masses. Histological analysis revealed cardiomyocyte hypertrophy in all mice receiving experimental diets (Table 2; Figure 1). An increase in cardiomyocyte diameter was statistically significant in mice fed a diet enriched with SFA (*p* < 0.001), 14:1 (*p* < 0.001), and 5:1 (*p* < 0.001), whereas a normal linear arrangement of myofibrils was found in all analyzed groups. Interstitial and perivascular fibrosis, as determined by the percentage of collagen, was statistically different between the SFA (*p* = 0.017) and 14:1 (*p* = 0.022) groups (Table 2; Figure 1). No significant statistical differences were demonstrated between the STD and experimental groups for blood plasma biochemical parameters (Table 2). 

### 3.2. Analysis of Heart Proteome

Bioinformatic analysis revealed 365–404 protein spots per each analyzed 2-D gel, representing protein profiles of murine heart. The coefficient of variation (CV) was estimated at the level of 46.62%, 44.82%, 43.77%, and 46.30% for the STD, SFA, 14:1, and 5:1 group, respectively. Of the analyzed protein spots, 285 were common to all gel members.

**Figure 2:**
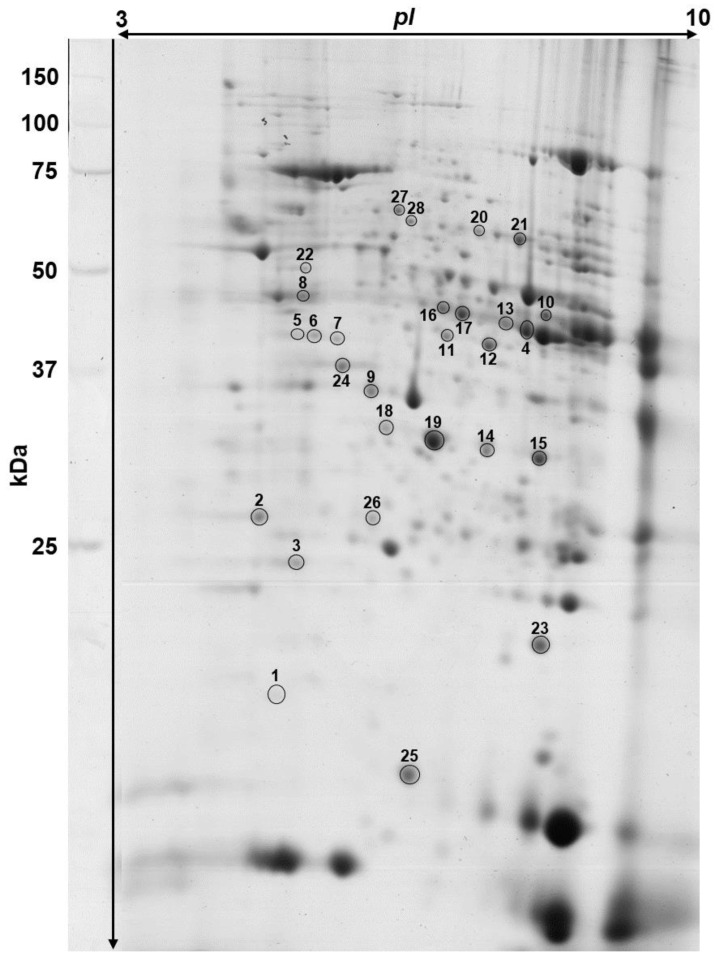
Representative two-dimensional electrophoresis gel image of the mouse heart proteome. Differentially expressed proteins between the animals fed standard diet (STD) and experimental high fat diets are indicated by numbers. Protein spot numbers refer to those presented in the heat map (Figure 3) and Table 3.

**Figure 3:**
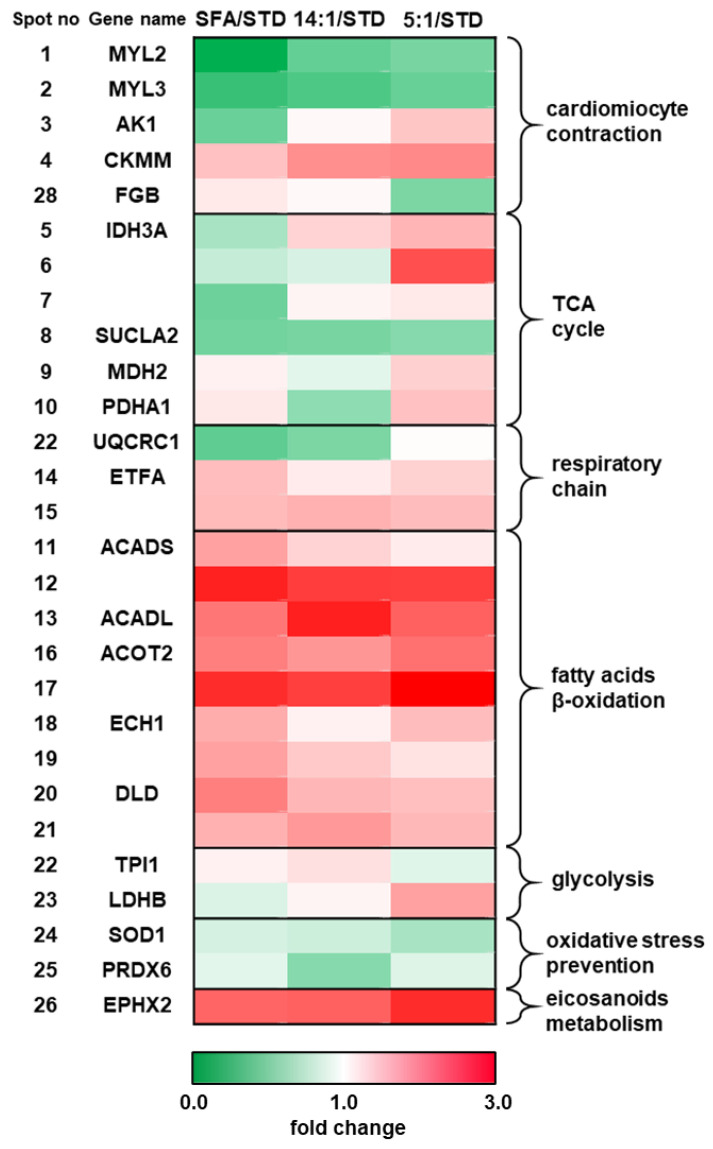
Heat map representing the identified proteins according to the magnitude of fold-change in the myocardium of mice fed high-fat diets with different fatty acid composition (SFA, n6/n3 PUFA 14:1, n6/n3 PUFA 14:1) compared to group of mice fed a standard diet (STD). Proteins were grouped according to their involvement in biological processes. Spot numbers refer to those presented on the 2-D proteome map (Figure 2) and Table 3. TCA - tricarboxylic acid cycle.

Comparative analysis demonstrated that a HFD based on SFA caused significant (*p* < 0.05) differences in the expression of 17 protein spots of the cardiac muscle proteome in comparison to animals fed the STD diet. Of these, 11 were upregulated and 6 downregulated in the SFA group. A high-fat diet rich in PUFA with the n-6/n-3 ratio of 14:1 was shown to induce significant expression changes of 14 protein spots, of which 10 were upregulated and 4 were downregulated in comparison to the STD group. Seventeen protein spots were significantly altered in the group of animals fed a high-fat diet enriched with PUFA with the n-6/n-3 ratio of 5:1. Of these, 14 were found to be upregulated and 3 were downregulated when compared to the STD group. Detailed data concerning the differences in protein expression and protein identification are presented in Table 3. Significantly expressed proteins are shown in the representative protein profile of cardiac muscle—Figure 2. The heat map summarising the expression changes of significantly altered protein spots in animals fed different HFDs in comparison to the STD group are given in Figure 3.

**Figure 4:**
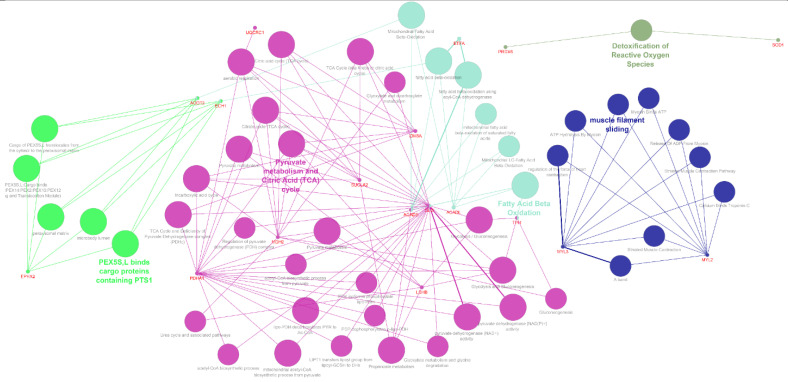
The ClueGO gene network of significantly altered pathways in cardiac muscle after 3 months of high-fat diets ingestion. Each node represents the Gene Ontology (GO) biological process/pathways and colors represent the GO group.

**Figure 5:**
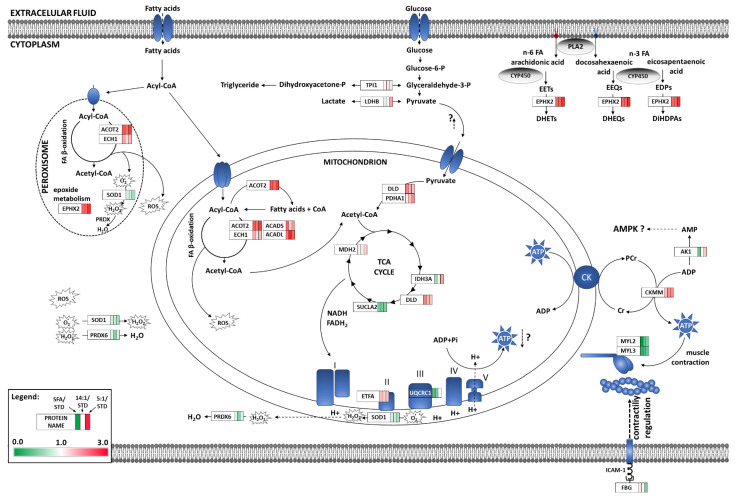
Overview of the effects of high-fat diets on the cardiomyocyte proteome. Identified proteins are shown according to the magnitude of fold-change in comparison to the STD group; red indicates upregulated proteins and green downregulated proteins in the myocardium of mice. EETs—epoxyeicosatrienoic acids; DHETs—dihydroxyeicosatrienoic acids; EEQs—epoxyeicosatetraenoic acids; DHEQs—eicosatetraenoic acids; EDPs—epoxydocosapentaenoic acids; DiHDPAs—dihydroxydocosapentaenoic acids; AMPK—AMP-activated kinase.

Significantly altered proteins were analysed using the Cystoscape software and the results of ClueGo enrichment are presented in Figure 4. Differentially expressed proteins were assigned to biological processes based on the aforementioned results as well as the data obtained from the STRING software analysis and uniport database. The groups of biological processes included: lipid metabolism and FA β-oxidation (ACADL, ACADS, ACOT2, ECH1, and DLD), glycolysis (TPI1 and LDHB), TCA cycle (IDH3A, SUCLA2, MDH2, and PDHA1), respiratory chain (ETFA and UQCRC1), myocardium contractility (MYL2, MYL3, AK1, CKMM, and FBG), oxidative stress (SOD1 and PRDX6), and PUFA eicosanoid metabolism (EPHX2). Contribution of the identified proteins to cardiomyocyte metabolism and contractile activity for each HFD is displayed in Figure 5. 

**Figure 6:**
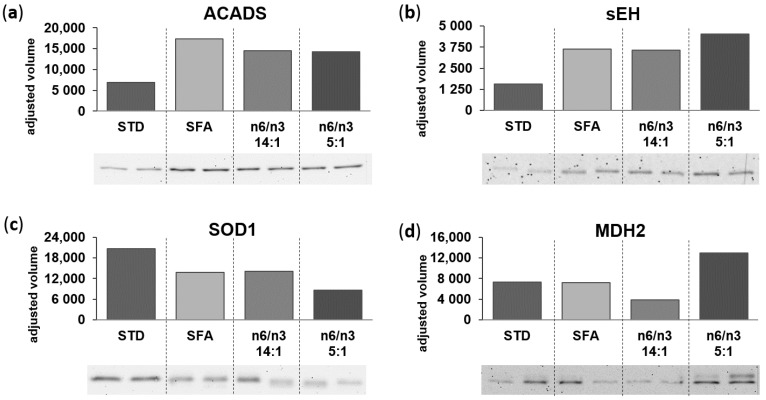
Validation of proteomic data obtained using 2-DE combined with MALDI-ToF MS. Protein expression levels of (**a**) short-chain specific acyl-CoA dehydrogenase, mitochondrial—ACADS (**b**) bifunctional epoxide hydrolase 2—sEH (**c**) superoxide dismutase 1—SOD1 (**d**) malate dehydrogenase, mitochondrial—MDH2. Western blots represent the murine myocardium from the control (STD) and experimental groups: SFA, 14:1 and 5:1.

Western-blot analysis was performed to verify the data concerning protein expression patterns based on 2-DE and subsequent identification using MALDI-ToF MS. We have selected proteins involved in fatty acid β-oxidation (ACADS), pyruvate metabolism and tricarboxylic acid cycle (MDH2), ROS detoxification (SOD1), and PUFA metabolism (EPHX2) for further validation based on the ClueGo analysis; the results of Western-blots are presented in Figure 6a–d, respectively. Expression patterns of selected murine heart proteins, confirmed by Western blot data, were consistent with 2-DE results.

## 4. Discussion

The present study aimed to analyse the effect of a three-month feeding with three types of high-fat diets (HFDs) containing different fatty acids (FAs) using proteomic and histological analysis of murine hearts. Our results clearly indicated that all HFDs, regardless of FA composition, promoted the development of obesity, as both weight gain and visceral fat mass were found to be significantly higher compared to control. It has been previously shown that diet-induced obesity is a key factor leading to histopathological myocardial changes associated with increased collagen deposition, which in turn may result in severe cardiac dysfunction [21]. This was in line with our findings as mice fed HFDs rich in SFA and PUFA with the n-3/n-6 ratio of 14:1 showed mild interstitial and perivascular collagen deposition. Furthermore, histological analyses of the heart also revealed signs of hypertrophy in response to all experimental diets. It should be noted that the highest hypertrophic lesions were observed in the SFA-fed animals, whereas the lowest were found in the group of mice fed a diet with the n-6/n-3 PUFA ratio of 5:1. As previously described and partially confirmed in our study, high fat diets rich in SFA [22,23,24] or diets based on equal proportions of SFA, MUFA, and PUFA [25] induced the development of cardiac hypertrophy and intravascular fibrosis. Additionally, excess dietary linoleic acid (LA) has also been related to increased collagen deposition in the left ventricle in C57BL/6 mice [16]. Collectively, our data may provide further evidence that high calorie intake triggers metabolic disorders, contributing to cardiac structure and function disorders [26]. The above-mentioned changes were also accompanied by cardiac protein expression alterations, including those related to cardiomyocyte contractility [27]. The results of the current study supported previous findings, as decreased expression of both isoforms of cardiac myosin light chains (MYL2 and MYL3), which are part of the cardiac contractile apparatus [28], was observed in all experimental groups. However, it should be emphasized that the most spectacular changes were observed in the SFA group, while the intake of HFD rich in PUFA led to less pronounced downregulation of MYL2 and MYL3 proteins. Myosin essential light chains play important roles in the regulation of cardiac myosin dynamics and crossbridge kinetics [29]. Attenuation of MYL2 phosphorylation is associated with the development of left ventricular hypertrophy resulting from depressed fractional shortening [30]. A marked reduction in MYL2 and MYL3 protein and mRNA levels was also observed in the hearts of rats with isoproterenol-induced cardiac hypertrophy [31]. Taking this into account, it can be assumed that HFDs, especially SFA-rich diet, led to impairments in the heart contractile apparatus, as indicated by decreased cardiac MYL expression, and could be one of the possible mechanisms for cardiac hypertrophy development.

There are some data that myocardial interstitial and perivascular fibrosis as well as cardiomyocyte hypertrophy may be related to increased expression of soluble epoxide hydrolase (sEH). Interestingly, our results demonstrated that mice fed all HFDs showed increased cardiac sEH protein expression compared to the STD diet. It should be pointed out that this protein plays a central role in the metabolism of bioactive lipid signalling molecules, as it is involved in the enzymatic conversion of omega-6 PUFA metabolites, epoxyeicosatrienoic acids (EETs) to less bioactive dihydroxyeicosatrienoic acids [32]. Accumulating evidence suggests that EETs display a broad spectrum of cardioprotective effects in the heart, including the impact on cardiac vasculature, heart fibroblasts and cardiomyocytes [33]. Recently, EETs have also been shown to cause coronary artery dilation, resulting in improved coronary blood flow [34], and to markedly limit collagen deposition [35]. A direct influence of EETs on cardiac myocytes have also been proposed, including the improvement of mitochondrial function, protection from angiotensin II-induced cardiac hypertrophy [36,37] and anti-arrhythmic effects [32]. Moreover, sEH is involved in the transformation of n-3 PUFA metabolites (epoxyeicosatetraenoic acids, EEQs, and epoxydocosapentaenoic acids, EDPs), exerting more potent anti-arrhythmogenic and anti-inflammatory effects than EETs to their biologically inactive forms [32,38]. Surprisingly, the highest expression of sEH in the present study was observed in animals fed a diet with the n-6/n-3 PUFA ratio of 5:1; however, this difference was not statistically significant. Furthermore, the increased expression of cardiac sEH protein, recorded in all experimental groups, was not accompanied by severe hypertrophic and fibrotic lesions, which may be attributed to the higher synthesis of PUFA derivatives known to exert cardioprotective functions. This is particularly interesting in view of recent data suggesting that cytochrome P450 (CYP) enzymes more efficiently convert n-3 acids to epoxy and hydroxy metabolites that potentially mediate beneficial cardiovascular effects [32]. The increase of sHE in animals fed the SFA diet was not surprising and was consistent with the results of Pakiet et al. (2020), who have reported an increase in cardiac PUFA concentration in mice fed a HFD rich in SFA and MUFA [39]. 

Fibrinogen is a thrombin coagulable glycoprotein circulating in the blood, and its degradation products are involved in the regulation of cardiac muscle functions [40]. In the present study, we observed a significant decrease in FGB expression in the group of animals fed a HFD with n-6/n-3 PUFA 5:1. It was previously shown that fibrinogen gamma chain (FBG) could depress cardiac muscle contractility after binding to intercellular adhesion molecule-1 (ICAM-1) [40]. The expression of myocardial membrane-bound ICAM-1 has been shown to increase in response to stress and cardiac dysfunction [41], as well as after a high-fat diet intake [22]. However, as previously shown by Yamada et al. (2008), n-3 fatty acids decreased plasma concentrations of soluble ICAM-1 in patients with metabolic syndrome [42]. Therefore, the decreased cardiac FBG expression in the group of mice fed the PUFA-rich diet with the low n-6/n-3 ratio, observed in the current study, might be explained by reduced ICAM-1 expression. However, this remains speculative and requires further research.

The contractility of cardiac muscle is strictly dependent on proteins involved in ATP regeneration such as creatine kinase (CKM) and adenylate kinase 1 (AK1). The activity of those proteins is crucial for maintaining energy homeostasis and contractile activity of cardiomyocytes [43]. CKM is responsible for energy accumulation in the form of phosphocreatine (PCr) or reverse ATP regeneration from PCr. In the present study, a notable upregulation of CKM was observed in mice fed the HFD enriched with PUFA. An increasing trend of CKM expression was also observed in the group fed the SFA diet; however, this observation was not statistically confirmed. This was consistent with previous findings of Rayner et al. (2020), who demonstrated enhanced CKM activity in the heart of obese individuals, which was considered a compensatory mechanism to maintain ATP delivery despite a reduced phosphocreatine-to-ATP ratio [44]. Moreover, elevated CK activity is also considered as one of the risk factors of heart failure in mice [45]. Therefore, it seems that the compensatory increase in CKM expression, observed in the present study, could reflect a lower myocardial ATP synthesis. This was further supported by significant changes in the expression of adenylate kinase 1 (AK1), a protein known to catalyse ATP resynthesis from ADP particles. An increased synthesis of this signalling molecule regulates cellular energy balance, i.e., via the stimulation of AMP-activated protein kinase (AMPK), which is involved in the induction of fatty acid β-oxidation in cardiomyocytes [46,47]. It was previously shown that metabolic stress, e.g., fatty acid overload, increased adenylate kinase (AK1) activity [43]. Interestingly, the direction of AK1 expression differed significantly between the SFA and PUFA 5:1 groups. This protein in mice fed the SFA diet was downregulated, whereas cardiac AK1 overexpression was demonstrated in animals fed HFD with the n-6/n-3 ratio of 5:1 compared to the control group. This could suggest enhanced cardiac AMPK activation in animals fed with the highest amount of n-3 FA. This is particularly interesting due to the fact that AMPK is involved in the heart failure prevention via the activation of FA β-oxidation in the failing heart, mitochondrial biogenesis, improvement of glucose utilisation, prevention of autophagy, and improvement of heart contractility. Moreover, AMPK activation is one of the therapeutic targets in improving failing heart function [48]. However, this phenomenon needs to be better understood, and thus requires further research. Nevertheless, we have shown here that ingestion of diets rich in n-3 PUFA may potentially trigger changes in cardiac AMPK activity.

One of the metabolic consequences of dietary FA overload is increased activity of intracellular enzymes responsible for metabolization of these energetic substrates. Peroxisomal and mitochondrial β-oxidation are the major degradation pathways in fatty acid metabolism [26,49]. A recent study of Sikder et al. (2018) showed an increased rate of FA β-oxidation in the hearts of mice subjected to diet-induced obesity [50]. In addition, the results of the present study demonstrated higher expression of myocardial proteins involved in lipid metabolism and FA β-oxidation in response to all experimental HFDs, regardless of FA composition. On the other hand, previous studies demonstrated that intense FA β-oxidation, observed in response to dietary FA overload, resulted in suppressed glycolysis and pyruvate oxidation [49,51]. Our results, however, did not confirm the latter findings as we recorded a significant increase in the expression of cardiac L-lactate dehydrogenase B chain (LDHB) in mice fed a diet rich in PUFA with the n-6/n-3 ratio of 5:1. This could suggest a significant conversion of pyruvate to lactate in this group of animals. It has been previously reported that cardiomyocytes undergo hypertrophic changes due to elevated LDH activity, which stimulates NDRG3 expression by increasing lactate generation, followed by cell growth in animals with metabolic disorders [52]. These compensatory mechanisms are activated in order to prevent heart failure [52]. In our current study, less pronounced cardiac hypertrophy signs demonstrated in animals fed the 5:1 diet, which was characterized by a higher n-3 PUFA content, led to the assumption that this effect was due to the increased myocardial LDHB expression.

Our further analysis showed increased expression of enzymes engaged in FA β-oxidation, accompanied by significant changes in the relative abundance of proteins of the TCA cycle and electron transport chain (ETC) in the myocardium of mice fed all HFDs. This could indicate impairments in mitochondrial ATP production. Our data were consistent with the study of Vileigas et al. [26], who reported significant changes in the expression of many rat myocardial TCA enzymes and ETC proteins that contribute to disturbances in ATP synthesis in response to a high-calorie westernized diet. The decrease in the ATP synthesis rate in the heart of mice, as a metabolic response to the westernized diet was also confirmed by Luptak et al. [53]. Interestingly, our research revealed increased expression of isocitrate dehydrogenase [NAD] subunit alpha (IDH3a) in the hearts of animals fed the SFA-rich diet, whereas this protein was shown to be downregulated in mice fed the PUFA-diet with the n-6/n-3 ratio of 5:1 compared to the STD group. The studies aimed at identifying heart metabolic shifts in a diet-induced pre-diabetic mice model [54] and type 2 diabetes [55] showed significantly downregulated IDH3a expression. Moreover, the same effect was observed in the murine heart following the onset of the pressure overload heart failure [56] and in failing heart [57]. This could suggest that IDH3a protein expression pattern in animals fed different HFDs in our study could reflect increasing TCA impairments, which in turn was associated with the observed hypertrophic changes.

High-fat diets rich in PUFA with the n-6/n-3 ratios of 5:1 and 14:1 caused significant downregulation of enzymes preventing oxidative stress such as SOD1 and PRDX6. However, the remaining HFDs triggered an opposite trend in the expression of the aforementioned cardiac proteins. SOD1 and PRDX6 are proteins involved in ROS neutralization, thus their expression is induced in response to increased ROS generation. Based on this, downregulation of those proteins may reflect a possible mechanism of reduced antioxidant defence in murine hearts in response to HFD-feeding. In our opinion, this effect was probably caused by enhanced ROS generation, resulting from intensified fatty acid β-oxidation. Intracellular ROS increase, with a concomitant suppression of the mechanism involved in antioxidant defence, may be responsible for the development of cardiac hypertrophy, cardiac fibrosis and subsequent contractility impairments [53]. Rich (2010) [58] showed that feeding C57BL/6 mice with a high-fat diet may have contributed to the impairment of cardiac defence mechanisms against oxidative stress due to significant SOD1 downregulation. Disruption of the mechanisms preventing oxidative stress, manifested by decreased expression of SOD1, SOD2, catalase, and glutathione peroxidase, both at the transcript and protein levels, was also observed in the hearts of rats fed high-fat and low-carbohydrate diets [59]. Moreover, a recent study of Vileigas et al. (2019) demonstrated that the expression of cardiac SOD1 was enhanced, while PRDX6 expression was decreased in mice fed a westernized diet [26]. 

## 5. Conclusions

Taken together, our data indicate that mice fed the HFD rich in SFA manifested the most severe cardiac hypertrophy and fibrosis lesions, whereas less pronounced changes were observed in the group of animals that ingested the highest amount of omega-3 FA. In general, all HFDs, regardless of FA composition, induced a comparable pattern of cardiac protein changes. However, it should be noted that three proteins, namely, IDH3A, LDHB, and AK1, were differently affected by different FA compositions. The high expression of these myocardial proteins found in the group of animals fed a HFD with the highest n-3 PUFA content could be closely related to the observed temporal delay in the development of hypertrophy. Future studies should pay particular attention to fully understand the role of AK1 and its potential involvement in AMPK activation in cardioprotection. In our opinion, soluble epoxide hydrolase (sEH) is another candidate protein for future functional analysis, as it is involved in the metabolism of cardioprotective n-6 and n-3 PUFA eicosanoids.

## Figures and Tables

**Table 1:** Components of experimental diets.

Group	Components	(g)	LA/ALA	% SFA	% PUFA	% MUFA
SFA	Labofeed H	790	1.41	76.87	11.04	12.09
	virgin coconut oil	200				
	pumpkin seed oil	10				
14:1	Labofeed H	790	13.76	1.68	82.21	16.10
	pumpkin seed oil	210				
5:1	Labofeed H	790	5.00	9.91	79.69	10.40
	sunflower seed oil	80				
	pumpkin seed oil	65				
	avocado oil	20				
	virgin coconut oil	20				
	hemp seed oil	15				
	corn oil	10				

LA/ALA—linoleic acid (LA, 18:2 n–6) to α-linolenic acid (ALA, 18:3 n–3) ratio; SFA—saturated fatty acids; MUFA—monounsaturated fatty acids; PUFA—polyunsaturated fatty acids.

**Table 2:** Morphological, histological and plasma biochemical parameters after three-month diets—standard (STD), experimental high-fat diets rich in saturated fatty acids (SFA), and rich in PUFA with the 14:1 n-6/n-3 and 5:1 n-6/n-3 ratios—expressed in arithmetic mean values (SD).

Parameter	STD	SFA	14:1	5:1
body weight (g)	35.03 ^A^	50.83 ^B^	47.49 ^B^	43.78 ^B^
visceral fat (g)	0.201 ^a^	0.311 ^b^	0.317 ^b^	0.306 ^b^
heart weight (g)	0.1871	0.2671	0.2384	0.2281
cardiomyocyte diameter (µm) *	8.19 (1.37) ^A^	14.46 ^B^ (3.36)	12.35 ^C^ (2.56)	10.03 ^D^ (1.64)
cardiomyocyte diameter range (µm)	4.95–13.21	8.01–19.81	7.35–16.98	6.13–16.84
collagenous tissue (%) *	5.42 ^a^ (1.48)	7.64 ^c^ (3.45)	7.32 ^bc^ (3.09)	5.92 ^ab^ (2.22)
lactate (mmol/l)	9.75 (2.48)	12.19 (3.13)	12.39 (3.42)	9.78 (1.79)
LDH (U/l)	1018.5 (451.9)	897.5 (229.7)	957.6 (444.8)	1021.1 (810)
CK (U/l)	233.7 (87.63)	150 (61.57)	183 (152)	289.1 (171)

With ^A, B, C, D^ significant differences (*p* < 0.01) between the groups are marked. The ^a, b, c^ was used to mark significant differences at *p* < 0.05 level. Values within the row marked with the different letters of alphabet differ significantly. With * the indices examined with Kruskal-Wallis test are marked. LDH -lactate dehydrogenase; CK—creatinine kinase.

**Table 3:** Differentially expressed protein spots between the standard (STD) and high-fat diet groups (SFA, 14:1, 5:1) in mouse cardiac muscle. Proteins were grouped according to their known biological processes. Spot numbers in the table correspond to the numbers in Figure 1 (2-DE proteome map) and Figure 3 (Heat Map).

Spot No	Protein Name	Gene Name	Seq. Cov. %	Mascot Score	STD	SFA	SFA/STD	14:1	14:1/ STD	5:1	5:1/ STD	Predicted pI/Mw	e\Estimated pI/Mw	SL
**Cardiac Muscle Contraction**														
1	Myosin regulatory light chain 2, ventricular/cardiac muscle isoform	MYL2	58	90	754.4	68.3 ^ab^	**0.09**	339.5 ^a^	0.45	392.3 ^b^	0.52	4.86/18.9	4.5/16.6	C
2	Myosin light chain 3	MYL3	61	146	1585.6	460.3	**0.29**	588	**0.37**	734.3	0.46	5.03/22.5	4.4/25.9	C
3	Adenylate kinase isoenzyme 1	AK1	52	108	45.4	21.2 ^ab^	**0.47**	48.3 ^a^	1.06	65.2 ^b^	**1.44**	5.67/21.6	4.6/24.8	C
4	Creatine kinase M-type	CKMM	36	101	264.5	390.5	1.48	494.6	**1.87**	506.2	**1.91**	6.58/43.2	7.6/41.60	C /MT
28	Fibrinogen beta chain	FGB	26	95	18.8	22 ^a^	1.17	20 ^b^	1.06	10 ^ab^	**0.53**	6.68/55.4	6.1/58.4	EX
**Glycolysis**														
23	Triosephosphate isomerase	TPI1	33	85	152	169.1	1.11	188.3 ^a^	1.24	134.7 ^a^	0.89	5.56/32.7	7.6/25.0	C
24	L-lactate dehydrogenase B chain	LDHB	37	108	161.3	139.9 ^a^	0.87	176.7 ^b^	1.09	278.1 ^ab^	**1.72**	5.70/36.8	5.3/36.3	C
**TCA Cycle**														
5	Isocitrate dehydrogenase (NAD) subunit alpha, mitochondrial	IDH3A	28	94	69.3	48.1 ^ab^	0.69	93.1 ^a^	1.34	108.9 ^b^	1.57	5.86/35.0	4.8/39.4	MT
6			39	121	31.4	24.7 ^a^	0.79	27.1 ^b^	0.86	73.7 ^ab^	**2.35**		5.0/39.6	
7			28	84	67	32 ^ab^	**0.48**	73.3 ^a^	1.09	78.5 ^b^	1.17		5.3/39.3	
8	Succinate-CoA ligase (ADP-forming) subunit beta, mitochondrial	SUCLA2	34	87	303.8	151.5	**0.5**	158.9	**0.52**	172	**0.57**	4.94/36.3	4.8/44.7	MT
9	Malate dehydrogenase, mitochondrial	MDH2	30	63	106.1	117.7	1.11	95.9 ^a^	0.9	144.8 ^a^	**1.36**	6.16/36.7	5.7/34.4	MT
10	Pyruvate dehydrogenase E1 component subunit alpha, somatic form, mitochondrial	PDHA1	22	81	156.8	184.5 ^a^	1.18	94.7 ^ab^	0.6	232.1 ^b^	**1.48**	8.49/43.9	7.4/42.2	MT
**Respiratory Chain**														
14	Electron transfer flavoprotein subunit alpha, mitochondrial	ETFA	40	96	92.8	140.4	**1.51**	107.9	1.16	125.4	**1.35**	8.62/35.3	7.2/30.6	MT
15			42	144	197	302.1	**1.53**	314.4	**1.6**	300	**1.52**		7.8/30.4	
22	Cytochrome b-c1 complex subunit 1, mitochondrial	UQCRC1	35	90	56.8	24.4 ^a^	**0.43**	30.1 ^b^	**0.53**	58.5 ^ab^	1.03	5.81/53.4	4.9/48.5	MT
**Fatty Acid Metabolism and β-oxidation**														
11	Short-chain specific acyl-CoA dehydrogenase, mitochondrial	ACADS	39	88	34.1	58.7	**1.72**	45.5	1.34	39.6	1.16	8.68/45.1	6.6/40.6	MT
12			49	156	53.7	145.6	**2.71**	134	**2.49**	133.2	**2.48**		7.2/39.7	
13	Long-chain specific acyl-CoA dehydrogenase, mitochondrial	ACADL	32	115	112.6	231.2	**2.05**	306.7	**2.72**	249.6	**2.22**	8.53/48.3	7.8/40.9	MT
16	Acyl-coenzyme A thioesterase 2, mitochondrial	ACOT2	40	142	87.2	173.2	**1.99**	157.7	**1.81**	182.9	**2.1**	6.88/49.9	6.6/43.7	MT
17			40	128	101.1	264.8	**2.62**	250.9	**2.48**	298.7	**2.95**		6.8/43.3	
18	Delta(3,5)-Delta(2,4)-dienoyl-CoA isomerase, mitochondrial	ECH1	34	74	21.6	35.3	1.64	23.9	1.11	32.9	**1.52**	7.6/36.4	5.9/31.7	MT/P
19			49	101	388.1	666.6	**1.72**	552.6	**1.42**	473	1.22		6.4/31.1	
20	Dihydrolipoyl dehydrogenase, mitochondrial	DLD	25	63	32.1	63.4	**1.98**	50.1	**1.56**	47.8	1.49	7.99/54.7	7.0/53.7	MT
21			28	90	104.4	166.9	**1.6**	186.4	**1.79**	161.9	1.55		7.6/56.3	
**Oxidative Stress Prevention**														
25	Superoxide dismutase (Cu-Zn)	SOD1	31	74	283.9	239.9	0.85	233	0.82	196.2	**0.69**	6.02/16.1	6.2/14.0	C/P
26/29	Peroxiredoxin-6	PRDX6	62	136	29.9	26.8	0.9	17.2	**0.57**	26.2	0.88	5.98/24.9	5.8/25.9	C
27/35	Bifunctional epoxide hydrolase 2	EPHX2	39	121	19.9	43.5	**2.18**	44.1	**2.22**	52.2	**2.62**	5.85/63.0	6.0/61.1	C/P

The results are the highest identification values from an average of two biological replicates. Statistically significant (*p* < 0.05) values of the average intensity of the SFA, 14:1 and 5:1 groups in relation to the STD group are marked in bold. With ^a,b^—significant differences (*p* < 0.05) between the animal groups fed with experimental diets (SFA, 14:1 and 5:1 groups) are marked. Significantly different walues are marked with the same letters of the alphabet. SL—subcellular localisation; C—cytoplasm; MT—mitochondrion; P—peroxisome; EX—extracellular.

## Data Availability

The data presented in this study are available on the reasonable request from the corresponding author.
